# Left Atrial Strain: Clinical Use and Future Applications—A Focused Review Article

**DOI:** 10.31083/j.rcm2305154

**Published:** 2022-04-26

**Authors:** Gergana Marincheva, Zaza Iakobishvili, Andrei Valdman, Avishag Laish-Farkash

**Affiliations:** ^1^Cardiology Department, Assuta Ashdod University MC, Ben-Gurion University of the Negev, 7747629 Ashdod, Israel

**Keywords:** left atrial strain, echocardiography, electroanatomical voltage mapping

## Abstract

Atrial cardiomyopathy represents a process of structural and functional changes affecting the atria and leading eventually to clinical manifestation of atrial fibrillation and risk of stroke. Multimodality imaging provides a comprehensive evaluation of atrial remodeling and plays a crucial role in the decision-making process in treatment strategy. This paper summarizes the current state of knowledge on the topic of left atrial strain imaging using two-dimensional speckle tracking echocardiography (2D-STE). We focus on our recently published data on left atrial remodeling assessed by 2D-STE versus high-density voltage mapping in patients with atrial fibrillation (AF).

## 1. Introduction

Atrial fibrillation (AF) is the most common arrhythmia, affecting up to 2% of the population [1] and is associated with significant morbidity and mortality. The substrate for AF lies in the process of left atrial (LA) remodeling, including LA fibrosis, fatty infiltration, amyloid deposition or LA dilatation, that lead to subsequent LA mechanical dysfunction and a delay in electrical conduction properties [2]. The term “fibrotic atrial cardiomyopathy” was introduced to describe these histological and pathophysiological changes.

The clinical approach in assessing patients with AF requires an evaluation of cardiac structure and function [1]. Such an assessment requires multi-modality imaging and influences our therapy strategy (rhythm control vs. rate control), need for ablation, pace and ablate approach, stroke risk stratification, and prognosis.

The assessment often begins with echocardiography as a first line diagnostic imaging strategy. The recent innovations in advanced cardiac imaging - cardiac magnetic resonance (CMR) and cardiac computed tomography (CCT) provide a comprehensive characterization of atrial anatomy. These imaging techniques are very accurate tools to exclude thrombus and to guide left atrial appendage (LAA) closure or catheter ablation (CA) of AF. In comparison to echocardiography, CCT provides better spatial resolution together with a rapid dataset acquisition. However, its disadvantages are the need to inject iodine contrast agent and radiation exposure. Another advanced imaging technique, CMR, provides very high temporal resolution, a unique feature of tissue characterization and no need for radiation exposure. However, its limitations are: long scan times, higher price, suboptimal reproducibility of results, and low availability. All these disadvantages drive the need for non-invasive, cheaper and widely available tools like echocardiography for evaluating atrial remodeling.

This paper summarizes the current state of knowledge on the topic of atrial strain imaging using two-dimensional speckle tracking (2D-STE). We focus on our recently published data on LA remodeling assessed by STE derived strain analysis versus electro-anatomical voltage mapping in patients with atrial fibrillation [3].

## 2. Assessment of Atrial Remodeling

Atrial remodeling is caused by collagen deposition in the cell interstitium followed by massive fibrosis formation [4]. This remodeling process causes alterations in the normal electrical conduction [5]. Fibrosis tends to increase progressively. Prevention of atrial remodeling is one of the treatment goals in order to delay disease progression.

The expert consensus in 2016 defined “atrial cardiomyopathy” as “any complex of structural, architectural, contractile or electrophysiological changes affecting the atria with the potential to produce clinically relevant manifestations” [6]. A working histological-pathophysiological classification was proposed with four different classes of atrial myopathy based on the predominant pathophysiological mechanism: (1) cardiomyocytes dependent changes; (2) mostly fibroblast dependent alterations; (3) mixed and (4) non-collagen deposits related.

Atrial remodeling pathophysiological process is defined by changes in the atrial structure and function resulting in atrial cardiomyopathy. LA remodeling is a complex process involving various interrelated pathophysiological mechanisms: (1) structural changes (LA dilatation) due to interstitial fibrosis; (2) functional remodeling (LA failure); and (3) electrical remodeling—changes in ion transport processes and action potential properties conducive for incident atrial fibrillation [7].

The identification of an advanced stage of fibrosis by multi-modality imaging can direct the therapy approach regarding intensity and need for invasive or conservative approach [8]. For example, the extent of fibrosis in the LA may guide the decision-making process in treatment strategy, selecting those patients suitable for ablation and may predict the long-term maintenance of sinus rhythm post AF ablation [9, 10]. It may also predict the risk of cardioembolic stroke and success after cardioversion in AF patients [11]. Moreover, the degree of fibrosis may guide the choice of AF ablation strategy [12].

### 2.1 Cardiac Computed Tomography (CCT) 

CCT has several potential roles in evaluating atrial remodeling in AF patients: CT provides accurate assessment of LA dilatation, which is a marker of AF progression [13]. In addition, CCT can detect LA wall infiltration by epicardial fat (location and volumetric assessment), which is a potential early marker for inflammation, for local slower activation time and for increased risk of AF recurrence after ablation [14]. Prior to AF ablation procedure, the pulmonary vein anatomy can be obtained by CT and LAA thrombus can be excluded; and finally, due to its great spatial resolution, multi-detector CT (MDCT) is able to accurately measure LA wall thickness, and thus, enable personalized AF ablation [15]. CCT can estimate LA volume with precision and with fast data acquisition, thus providing a more accurate evaluation of LA volume compared to TTE [16]. However, compared to echocardiography, its disadvantages are the need to inject iodine contrast agent and radiation exposure, and compared to LGE-CMR, it has a low contrast-to-noise ratio, limiting its ability to differentiate between normal tissue and scar.

### 2.2 Three-Dimensional Electro-Anatomical Mapping (3D-EAM) 

Cardiac imaging using 3D-EAM system, combines both anatomical structure and electrophysiological data and is capable to display hybrid information in a three-dimensional, visual way. Low atrial endocardial voltage zones (LVZs), measured by EAM system, represent a surrogate maker for the presence of atrial fibrosis and are targets for AF ablation [6]. Studies showed that those LVZs correlate with local conduction disturbances in the atrial tissue [17] and that AF ablation in patients with more than 5% of LVZs areas had lower procedure success with higher rate of AF recurrence [18]. However, intracardiac voltage mapping appears to be an invasive and expensive tool.

### 2.3 Cardiac Magnetic Resonance with Gadolinium Delayed-Enhancement (LGE-MRI) 

CMR represents valuable imaging method for atrial fibrosis identification [19]. The contrast enhancement technique is used for cardiac tissue characterization, specifically for assessment of myocardial scar formation and regional myocardial fibrosis [20]. Caixal *et al*. [21] recently reported interesting results showing accurate detection of LA fibrosis in cardiac late gadolinium enhancement magnetic resonance imaging (LGE-MRI). They showed a strong correlation between MRI detected fibrosis and endocardial voltage and conduction velocity using EAM.

The quantitative analysis of atrial fibrosis by MRI has been shown to be associated with the risk of stroke [11]. In addition, MRI analysis of LA fibrosis is currently being evaluated for decision making regarding selection of patients for AF ablation, and for planning and guiding AF ablation procedures. Marrouche *et al*. [10] established the Utah stage classification for a quantitative analysis of LA fibrosis (i.e., LA wall enhancement as a percentage of the total LA wall, stages I–IV), and showed that the extent of atrial fibrosis detected by LGE-MRI was independently associated with AF recurrence in patients undergoing AF ablation. However, the implementation of image-guided atrial fibrosis ablation did not significantly improve the success rate of the procedure compared to pulmonary vein isolation in treatment resistant AF, according to the early results of the randomized DECAAF II trial. Important advantage of using this image-integration method of ablation was noted in the subgroup of patients with early stages of fibrosis [22].

Although CMR is becoming more available, providing best soft tissue contrast compared to other techniques without radiation exposure, its limitations include long scan times (due to its dependency on heart movements and breathing and the need for multiple images), challenges in evaluating atrial fibrosis during AF rhythm due to irregularity; higher price; suboptimal reproducibility of results; and low availability in different medical centers.

### 2.4 Echocardiography

Every newly diagnosed patient with AF is usually referred to transthoracic echocardiography (TTE) for comprehensive evaluation of atrial structure and size, valvular anatomy and function, as well as LV systolic and diastolic performance [1, 23]. Doppler and volumetric approaches were the first methods used to assess LA function; a larger atrial volume was shown to be associated with a higher risk of AF in older patients [24]. LA volume index (LAVI) was recognized as a key prognostic marker [25] and has a central role in the multidisciplinary management of patients with AF diagnosis [26]. The Doppler obtained E/e’ ratio (a marker for left ventricular filling pressure) can be helpful to evaluate atrial compliance and LA stiffness.

More recently, speckle tracking echocardiography (STE), has been applied to assess LA remodeling and fibrosis. Left atrial STE derived strain imaging has appeared as a non-invasive and affordable diagnostic modality for the accurate detection of atrial cardiomyopathy, as opposed to the highly invasive 3D-EAM and CMR methodology with limited availability. It should be emphasized that left atrial fibrosis cannot be assessed directly on echocardiography, but studies have shown direct correlation between LA global strain measured by STE and the degree of LA fibrosis on histopathology [27].

## 3. Atrial Strain Using 2D-STE 

LA strain is an STE–derived analysis of LA mechanical function with the advantage of being tissue Doppler derived, angle-independent measure of atrial function. It allows the assessment LA mechanical performance in each phase of the cardiac cycle (reservoir, conduit, and contractile). The parameters acquired with this technique cannot be compared directly to conventional echocardiographic parameters, but when used in combination, LA strain can provide complementary information on structural remodeling and mechanical dysfunction of left atrium [28, 29].

The EACVI/EHRA Expert Consensus Document [30] for the evaluation of patients with AF, indicates STE and LA strain as valuable complementary tools in this setting and the new European AF guidelines [26] underscore the use of LA strain for more accurate assessment of LA function. The lack of large prospective studies and standardized methods for LA strain measurement, along with several technical and methodological challenges, encouraged the publication of the EACVI/ASE/Industry Task Force consensus document to standardize definitions and techniques for using 2D-STE [31], aiming to standardize the assessment of LA, right ventricle, and right atrial myocardial deformation.

### LA Mechanical Deformation is Divided into Three Phases

**Fig. 1.**
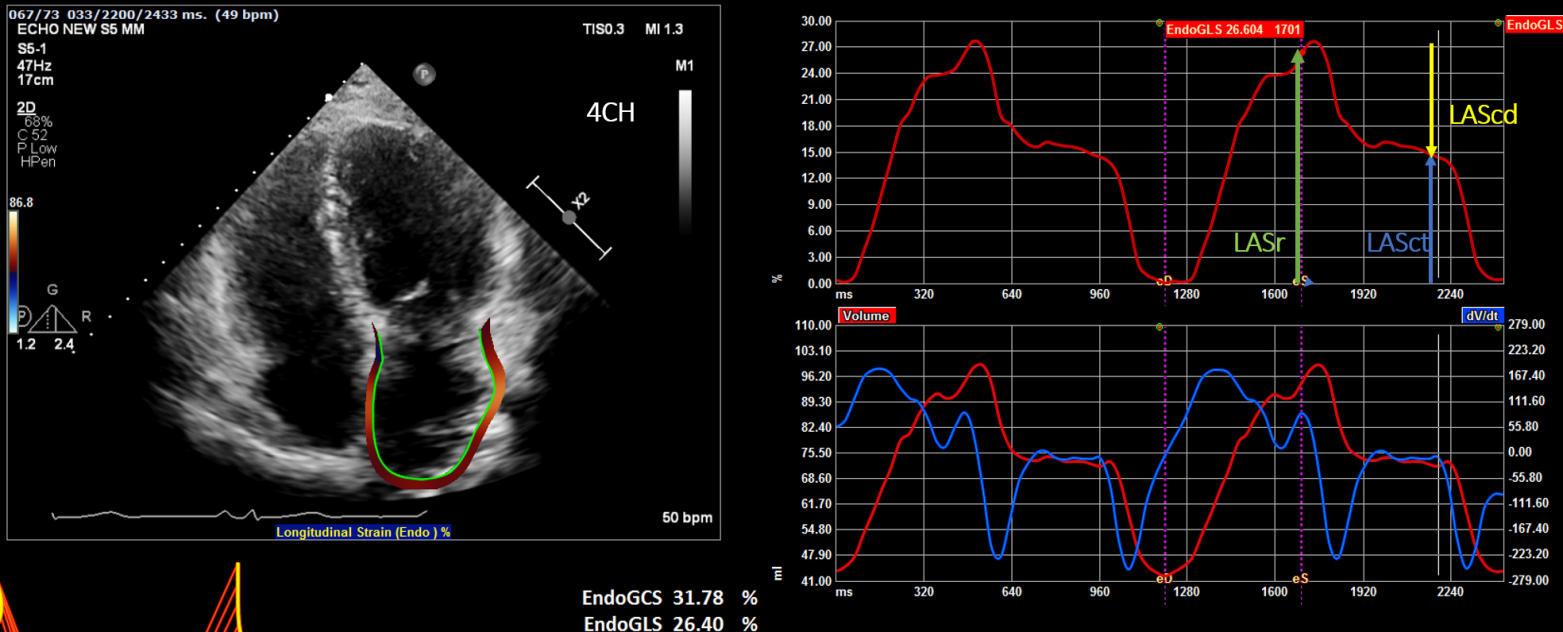
**Display of the left atrial deformation over a cardiac cycle, starting at the QRS as zero reference point**. The peak positive longitudinal strain (LAS) corresponds to atrial reservoir function (LASr — reservoir strain), strain during early diastole reflects atrial conduit function (LAScd — conduit strain) and strain during late diastole corresponds to atrial contractile function (LASct — LA contraction strain).

(a) Reservoir phase—LASr (left atrial reservoir strain): atrial diastole (occurs during ventricular systole)—begins at the end of ventricular diastole (mitral valve closure) and continues until mitral valve opening. Represents the time of LV isovolumic contraction, ejection and isovolumic relaxation (Fig. 1).

(b) Conduit phase—LAScd (left atrial conduit strain): passive atrial systolic phase during ventricle diastole. Starts with mitral valve opening through diastasis until the onset of LA contraction in patients with sinus rhythm. In patients with AF, it continues until the end of ventricular diastole (mitral valve closure).

(c) Contraction phase—LASct (left atrial contraction strain): starts with the onset of LA contraction until the end of ventricular diastole (mitral valve closure) in patients with sinus rhythm.

The importance of the reference frame of zero strain was emphasized in the EACVI/ASE/Industry Task Force consensus document. Choosing different options for zero strain reference affects LA strain measurements [31]:

(1) Zero strain reference set at left ventricular end-diastole-recommended (Fig. 1).

(2) Zero strain reference set at the onset of LA contraction.

The use of different zero reference point in previous research works led to different normative values [32, 33, 34]. Using the recommended end-diastole zero reference makes atrial strain analysis possible in all patients (including patents in AF), and it makes the analysis easier, since with this zero reference the LA strain measurement is equal to the first positive peak systolic value of the LA strain curve. LA reservoir function analysis by STE is the most commonly used LA strain parameter with the largest evidence supporting its prognostic value [30, 34]

A large-scale population-based study by Liao *et al*. [35], provided age- and sex- related reference values of LA deformation indices and proved the utility and feasibility of strain analysis in evaluation of LA function. Their findings were consistent with the Task Force efforts of the American and European echocardiographic societies to standardize LA strain measurements [30, 31].

In 2018, Sugimoto *et al*. [36], published echocardiographic reference ranges for normal left atrial strain analysis. The NORRE study provided contemporary, age-specific, applicable echocardiographic reference ranges, using vendor-independent software (VIS) system (TomTec Imaging System, Munich, Germany) for analyzing the data.

## 4. Clinical Implications of Atrial Strain

There is rapidly growing body of literature that supports LA strain use in different clinical settings.

### 4.1 Atrial Fibrosis—A Hallmark of AF

Myocardial fibrosis is the substrate of AF and its characterization could be used to guide our treatment approach.

Studies showed that 2D-STE obtained atrial strain measurements correlated directly with histologically proven fibrosis of the LA wall [27]. Kuppahally *et al*. [37] reported inverse correlation between the degree of fibrosis detected by contrast enhanced MRI and LA strain analysis in patients with persistent AF compared with paroxysmal AF.

Watanabe *et al*. [38] found correlation between EAM and 3D-speckle tracking echocardiography (STE) methods in paroxysmal AF patients during SR, even in patients with less advanced anatomic remodeling. This group showed that LA dyssynchrony is latent in patients with AF in the early remodeling phase, and that the early remodeling changes can be detected using 3D-STE. However, 3D-STE analysis is possible only during sinus rhythm and is limited by its lower accuracy and temporal resolution. To overcome these limitations, our group used 2D-STE derived LA strain instead of 3D-STE for the estimation of LA fibrosis grade [3]. An evaluation of LA reservoir strain (LASr) was chosen, since it represents atrial filling and compliance. Most importantly, LASr was shown to be less affected by the cardiac rhythm in the time of examination-can be performed during AF [31].

Similar to methods used in previous works [18], we defined low voltage zones (LVZs) as an area with voltage amplitude lower than 0.5 mV and covering more than 5% of LA area, measured by multi-electrode, contact force mapping catheter. This cut-off reflected the minimum amount of LA fibrosis detected by LGE-CMR in previously reported study [39].

We used this cutoff to compare patients with LVZ (≥5%) versus those without LVZ (<5%) in EAM regarding association with atrial LASr [3]. Patients with LVZs more than 5% area had lower atrial reservoir strain. Our study demonstrated negative correlation between the extent of atrial fibrosis measured by EAM and LASr measured by 2D-STE and suggested that non-invasive STE derived atrial strain imaging can be easily applied for evaluation of LA fibrosis, even in patients with irregular rhythm [40]. Strain analysis holds the potential for better prediction of ablation procedure success and better selection of patients who would benefit from it. Moreover, reduced atrial strain was associated with the progression from paroxysmal AF to persistent AF [41].

### 4.2 LA Strain in AF Therapy and Follow Up

LA strain measurements are complementary to volumetric measures, but are more sensitive, because they can detect LA dysfunction, before LA dilatation occurs [42, 43]. In a study by Cameli *et al*. [43], there was a strong association between LASr and cardiovascular events after adjustments, and both LA emptying fraction and LASr were superior and incremental to LAVI, suggesting that impaired reservoir function (LASr) may be a more sensitive indicator of left atrial remodeling [44, 45]. Studies have shown an association between reduced LASr and contractile function, as well as paroxysmal AF that precedes LA enlargement [46, 47]. Furthermore, LASr at baseline, prior to ablation, has been shown to be an independent predictor of LA reverse remodeling [48, 49]. In patients undergoing AF ablation, LASr predicts the maintenance of sinus rhythm after ablation in both paroxysmal and persistent AF forms [28, 50, 51, 52].

Interestingly, LA peak longitudinal strain (LASr) results are reduced after electrical cardioversion or ablation, and tend to increase gradually in the following period of months after the procedure, suggesting an increased thromboembolic risk [53, 54]. These results are probably related to the atrial “stunning” after conversion to SR. Moreover, reduced atrial strain predicts arrhythmia recurrence post cardioversion during a period of six months follow up [55].

### 4.3 Cardioembolic Stroke 

Ischemic stroke is a leading cause of morbidity and mortality in patients with AF. The presence of AF increases significantly (fivefold) the risk of stroke, with the left atrial appendage thrombus formation the most common cause [56, 57]. The risk of stroke is in correlation with LA size [58]. Risk factors, such as age, obesity, diabetes, hypertension and sleep apnea are important factors in promoting atrial cardiomyopathy characterized by endothelial dysfunction, fibrosis and blood stasis. These changes create a prothrombotic milieu in the left atrium and have been related to the process of clot formation even before arrhythmia occurs.

Stroke prevention plays a major role in the management of AF patients and the decision for anticoagulation is currently based on clinical risk scores. Parameters of LA function are not implemented in the risk score. Several LA imaging-derived parameters have been shown to correlate with the risk of thromboembolism—LA dilation, spontaneous echo contrast (SEC) in LAA, LAA thrombus, reduced LAA velocity, 2D-STE reservoir strain (LASr) and LAA morphology (non-chicken wing) [59]. It is unclear however, if their addition to the current risk scoring system will improve the risk stratification for stroke.

The role of echocardiography in excluding thrombus in LAA and in characterizing LA enlargement in patients with AF is complemented by the evaluation of LA strain.

LA strain parameters have been shown to be associated with thromboembolic risk. LASr is associated with stroke among patients with permanent AF [60] and is negatively associated with cardioembolic risk in patients with paroxysmal and persistent AF [61]. LA strain also correlates with CHADS2 score [62].

LA strain has been suggested as being a surrogate marker of AF occurrence in patients with cryptogenic stroke [63]. In study by Leong *et al*. [64], patients with cryptogenic stroke and no history of AF demonstrated significantly lower left atrial reservoir strain compared to controls. LASr analysis provided incremental discriminatory value in the identification of stroke patients, with the ability to detect subtle LA dysfunction, before occurring of arrhythmia. The data support the hypothesis that remodeling and reduced atrial contraction predispose to subsequent thromboembolism [65] and emphasize the need for early recognition of subtle left atrial abnormalities.

Recently, Park *et al*. [66] reported, that reduced LA reservoir strain <14.5% was associated with an increased risk of stroke in patients with heart failure and sinus rhythm.

Furthermore, Sade *et al*. [67] showed that STE imaging evaluation of LA dysfunction by LA reservoir atrial strain and contraction (LASr and LASct) predicted both cryptogenic stroke in general and embolic stroke of undetermined source (ESUS), independently from LA volume index (LAVi) and CHA2DS2-VASc score. LASr >26% yielded 86% sensitivity, 92% specificity, 92% positive, and 86% negative predictive values for the identification of ESUS.

### 4.4 Left Atrial Strain in Heart Failure (HF) and Valvular Heart Disease

The crucial role of the LA in the pathophysiology of HF with reduced ejection fraction (HrEF) and HF with preserved ejection fraction (HpEF) and in valvular diseases is comprehensively exposed in the recent review article by Carpenito *et al*. [68].

LA enlargement represents compensatory response to longstanding rise in LA pressure and LV filling pressure in the early stages of diastolic dysfunction, so it can maintain stroke volume. Parallel to LA dilatation, structural alterations begin and fibrosis occur, until left atrium loses its contractile function and starts to behave like a conduit with subsequent mechanical and electrical failure [69]. When compliance is lost, reservoir and conduit atrial function are altered and further increase in pulmonary pressure reflects the underlying interaction between right ventricle and pulmonary circulation uncoupling.

There is a strong correlation between LA volume and its mechanical performance, but changes in LA deformation can occur earlier than cavity remodeling and dilatation, and LA strain has been shown to be potential marker of early identification of subclinical heart dysfunction [70].

These early changes in atrial compliance can be captured by atrial strain analysis, adding incremental information to the structural information provided by the conventional echocardiographic indices. Early detection of atrial remodeling with better understanding of LA function can guide the correct time to intervene, just before the final fibrotic changes occur.

Kurt and colleagues [71] reported reduced levels of LASr in patients with HFpEF compared to patients with LV diastolic dysfunction with no cardiac failure. LASr <23% has been associated with worse New York Heart Association (NYHA) functional class and elevated pulmonary capillary wedge pressure (PCWP) [72]. Recently, Khan *et al*. [73] reported results of a robust meta-analysis, summarizing studies of LA function in HFpEF. Volumetric measurements of LA function were lower in HFpEF patients compared to controls. Importantly, LA reservoir strain was associated with the composite of mortality and HF hospitalization.

Reduced reservoir function has been associated also with symptoms [74] and with peak oxygen consumption during cardiopulmonary exercise testing, even after adjustment for LV and right ventricular (RV) longitudinal strain [75].

The association of impaired LA reservoir and increased stiffness with abnormal exercise hemodynamics in HFpEF patients could provide significant diagnostic utility in elderly ambulatory patients with dyspnea [76].

Another study by Melenovsky *et al*. [77], demonstrated important differences in LA properties between HFrEF and HFpEF. Patients with HFrEF had larger LA volumes (LA volume index 50 versus 41 mL/$m^{2}$; *p *< 0.001), whereas HFpEF patients had higher LA peak pressures. These differences could be a reflection of different pathophysiological mechanisms in LA cardiomyopathy—HFrEF is characterized by greater eccentric LA remodeling, whereas HFpEF by increased LA stiffness, which might contribute to greater atrial fibrillation burden.

Further insights into the role of LA in functional response to heart failure were reported by the group of Sugimoto *et al*. [78], focusing on the role of LA as a capacitor in the interaction between LV dysfunction, pulmonary congestion, and RV dysfunction. A negative correlation was found between impaired LASr (<23%) and LA enlargement (>34 mL/$m^{2}$). In HFpEF patients atrial strain reserve was reduced slightly than in patients with HFrEF. The investigators also found an association between minute ventilation and carbon dioxide production (VE/${\mathrm{VCO}}_{2}$) slope—marker of excessive ventilation—with worse prognosis in HF. This parameter corresponded with the extent of elevation in LA pressure, suggesting that pulmonary pressure plays a key role in the relationship between LA strain and VE/${\mathrm{VCO}}_{2}$ [79]. Moreover, the authors evaluated RV–pulmonary artery (PA) coupling as the ratio between PA systolic pressure and tricuspid annular systolic excursion. This ratio increased in patients with HF, especially in HFrEF. The observations of this study are representing the close interaction between RV function and afterload—relating the RV remodeling to chronic pressure overload to failing LA [80, 81, 82].

Reddy and colleagues [83] demonstrated that LA compliance and mechanical performance decline with increasing AF burden in HFpEF, increasing the likelihood of AF progression. These changes in LA compliance may lead to development of specific type of HFpEF characterized by worsening pulmonary hypertension and right cardiac failure.

The same research group [84], demonstrated that atrial strain analysis (LASr) may improve diagnostic accuracy beyond conventional echocardiographic indices to differentiate HFpEF from non-cardiac causes of dyspnea (NCD). Of all echocardiographic parameters, LASr best discriminated HFpEF from NCD, outperforming E/e’, LA enlargement, tricuspid regurgitation velocity >2.8 m/s, left ventricular hypertrophy and left ventricular global longitudinal strain. Indexing LASr to estimated LA pressure (E/e’) as a surrogate for LA compliance further improved diagnostic performance.

Moreover, several studies using strain analysis revealed that significant structural and electrical reverse remodeling of LA can occur after reducing LA pressure and overload, highlighting LA strain as a potential therapeutic target [85]. Reverse remodeling in LA, as detected by strain analysis, can occur after many therapeutic modalities: medical or resynchronization treatment for HF; ablation or cardioversion for AF; and also after valvular repair or replacement for valvular heart disease [86, 87].

## 5. Limitations

Important issues regarding LA strain analysis are under discussion:

Atrial reservoir function is defined by the positive peak of atrial strain curve (LASr), during the maximum elongation of the LV in ventricular systole. For this reason, LASr reflects also the longitudinal contraction of the LV chamber in the same time period of the cardiac cycle. We assume that LASr is an intrinsic performance measurement of the LA myocardium during ventricular systole, and therefore on physiological grounds should be associated with extent of LVZs of fibrosis. In fact, the deformation of the LA during ventricular systole is largely, if not entirely, the result of LV contraction and the traction the LV exerts on the LA as its longitudinal dimension shortens within the pericardial sac. Thus, it is possible that reduced LV (and not LA) longitudinal strain during systole is primarily responsible for our measurements [88].

Barbier *et al*. [89], demonstrated that the systolic descent of the LV base has a significant influence on the LA deformation during reservoir phase and that acute LV regional ischemia in their pig model, increased LA stiffness and impaired LA reservoir function by reducing LV base descent.

Later on, Russo and colleagues [45] demonstrated that a reduction in longitudinal strain of LV (found to be an independent predictor of incident AF) can be in fact associated with a lower LASr.

Indeed, global longitudinal strain of LV function has not been assessed comprehensively in the studies using LA longitudinal strain analysis. However, the vast majority of AF cases are the result of LV dysfunction, such a finding would be reasonable, as was demonstrated by Russo and colleagues [45].

On the contrary, Cameli *et al*. [70] demonstrated that LASr was independent from LV strain in identifying heart dysfunction in an earlier stage. Therefore, the issue whether LASr is influenced by the LV function remains controversial.

Instead of focusing on the LA reservoir function, focus on contractile strain analysis has been suggested and worth consideration in future research studies [90].

## 6. Future Perspectives

The development of LA strain imaging reveals its potential role in the evaluation of LV diastolic function, adding incremental information to the data obtained with conventional echocardiographic indices [91]. A strong association of reduced LV filling pressure with subsequent LA reverse remodeling and improved performance, has been demonstrated by strain analysis, suggesting future utility in clinical practice.

Numerous studies have been published on the role of atrial strain imaging in the specific setting of heart failure. There are still difficulties in characterization of certain types of HFpEF and LA strain analysis may be useful in clarification of this topic. LA strain could also be useful in studying the effect of drugs on atrial remodeling in patients with cardiac failure.

LA strain imaging implementation in the research and clinical management of AF is accumulating. Increasing number of studies are supporting LA strain utility in assessment of atrial remodeling process, its association with thromboembolic risk, AF ablation success and arrhythmia recurrence. Larger studies are needed to confirm this association and to investigate whether strain analysis can be applicable to identify patients at risk in everyday clinical practice.

Moreover, new efforts to perform analysis of atrial strain without depending on different vendors and using new algorithms are emerging.

## 7. Conclusions

In the era of precision medicine, personalized approach based on multimodality imaging information to every specific patient is more and more accentuated, and strain analysis will potentially have a major contribution in this regard. Use of LA strain in the comprehensive assessment of patients with AF, HF of different types, and valvular heart disease may fill the gap of affordable, easy to use method of risk stratification and precise timing of therapeutic intervention.
